# New fabrication method for di-indium tri-sulfuric (In_2_S_3_) thin films

**DOI:** 10.1038/s41598-022-11107-w

**Published:** 2022-04-29

**Authors:** Ahmed I. Ali, Medhat Ibrahim, A. Hassen

**Affiliations:** 1 Basic Science Department, Faculty of Technology and Education, Helwan University, Cairo, 11281 Egypt; 2 Nanotechnology Research Center (NTRC), British University in Egypt (BUE), Elshrok-City, 11837 Cairo Egypt; 3 Department of Physics, Faculty of Science, Fayoum University, El Fayoum, 63514 Egypt; 4 Molecular Spectroscopy and Modeling Unit, Spectroscopy Department, National Research Centre, 33-El bohouth St., Dokki, 12622 Giza Egypt

**Keywords:** Surfaces, interfaces and thin films, Structure of solids and liquids

## Abstract

Di-indium tri-sulfuric (In_2_S_3_) thin films are fabricated with annealing indium thin films in a sulfur environment. The effect of both annealing temperature and pressure on the structure, morphology, Raman, and photoluminescence (PL) spectroscopy has been studied. The X-ray diffraction (XRD) and field emission scanning electron microscopy (FE-SEM) of the prepared thin films showed different structural phases and morphology with varying annealing temperature and pressure. Energy dispersive X-ray (EDX) analysis confirmed the chemical composition and the atomic ratio of In/S for the In_2_S_3_ thin films. The optimum annealing conditions of In_2_S_3_ thin films are 550 °C and 100 Torr. The outcome results revealed a new good growth method for In_2_S_3_ thin films to be used for different applications.

## Introduction

Layered materials such as graphene, black phosphorus, and layered transition metal dichalcogenides (TMDs) have attracted researchers owing to their unique structure, novel physical properties, and potential applications^1^. In this sense, MoS_2_ is potentially important in optoelectronics because its bandgap is in the visible light range. MoS_2_ is a direct transition bandgap in the case of the monolayer, while it is varied to indirect transition with bilayers, thin film, and bulk form^2–4^. Di-indium tri-sulfuric (In_2_S_3_) is known to be layered with hexagonal, tetragonal, and spinal structures. One of the most unique feature properties of In_2_S_3_ is the bandgap energy. It is a layer-independent type. In addition, In_2_S_3_ is considered a promising semiconductor material for many applications including; optoelectronic, photovoltaic, and photoelectrochemical solar cells. The reasons behind the potential applications of In_2_S_3_ are its stability, transparency, wider bandgap energy, and photo conducting behavior^5,6^. In_2_S_3_ exhibits different phase structures like α, β, and γ depending on the preparing conditions^7^. The most stable phase among them at room temperature is the β-In_2_S_3_ phase which was found to be a stable crystalline phase with a tetragonal structure^8^. Moreover, In_2_S_3_ is a nontoxic material of n-type semiconductor with an energy band gap of 2.0–2.8 eV, depending upon the preparation technique^9–11^, and having a high transmittance in the visible part of the spectrum.

Many routes of preparation strategies had been applied for getting high-quality In_2_S_3_ thin films, that were successfully synthesized using numerous techniques such as close space evaporation^11^, thermal evaporation^12,13^, chemical bath deposition^14,15^, physical vapor deposition^16^ and spray pyrolysis^17,18^. It is found that the physical properties of the In_2_S_3_ are strongly dependent on both the deposition technique and deposition conditions such as temperature and pressure, as well as annealing time^19–25^. Recently, Sim et al.^26^ reported on the photoluminescence (PL) of In_2_S_3_ thin films. They prepared their films on SiO_2_ substrate using physical vapor deposition. The strong PL efficiency at room temperature is attributed to the oxygen isoelectronic impurity that substituted sulfur.

It is a good motivation to fabricate the indium thin film with the thermal evaporator method and anneal in a sulfur environment using the chemical vapor deposition (CVD) method. In this way, we optimized the annealing conditions such as temperature, pressure, time, and gas. It was found that CVD is the most suitable method for growing In_2_S_3_ thin films. This technique is simple and cheap compared to the previously mentioned techniques currently in use in photovoltaic device fabrication. It was able to deposit large area films in uniform surface morphology using this simple technique. Moreover, it is easy to change the atomic concentrations in the as-prepared films. This is useful in controlling the electrical and optical behaviors of the thin films by changing the atomic ratio of In and S atoms (In/S).

Based upon the above consideration, this work aimed to add a new fabrication technique for In_2_S_3_ thin films. Two deposition steps were used; evaporation of indium metal using a thermal evaporator, and annealing indium thin films in the sulfur environment in the two-zone furnace. Different preparing conditions such as annealing temperature and pressure have been studied. The structure and morphology of In_2_S_3_ were investigated using XRD and FE-SEM. Also, PL and Raman spectroscopy were detected and discussed for all synthesized thin films. The synthesis conditions of In_2_S_3_ thin films with the ability to change the structure, morphology, Raman, and PL spectroscopy are discussed and explained.

## Materials and methods

Before deposition, n-type SiO_2_/Si substrates were supersonically cleaned successively in acetone, ethanol, and distilled water for 30 min to remove any organic compound and other ionic impurity elements. Then, silicon wafers were dried at N_2_ atmosphere. Around 0.30 g of pure sulfur powder (99.99%, Alfa) was placed in a graphite crucible. The evaporator deposited indium films were kept over a silicon wafer (SiO_2_/Si) and were placed 20 cm far from the sulfur powder in the quartz tube. Generally, the In_2_S_3_ films were synthesized in two deposition steps namely: the first step: evaporating indium metal on SiO_2_/Si-substrate using the thermal evaporation technique. The films are grown under vacuum at a gas pressure of 1 × 10^–4^ Pa using a tungsten boot. Optimum conditions have been done to get homogeneity indium films. We fixed the deposition conditions for indium thin films to keep all films with the same conditions such as time, thickness, roughness, color, etc.

**Figure 1:**
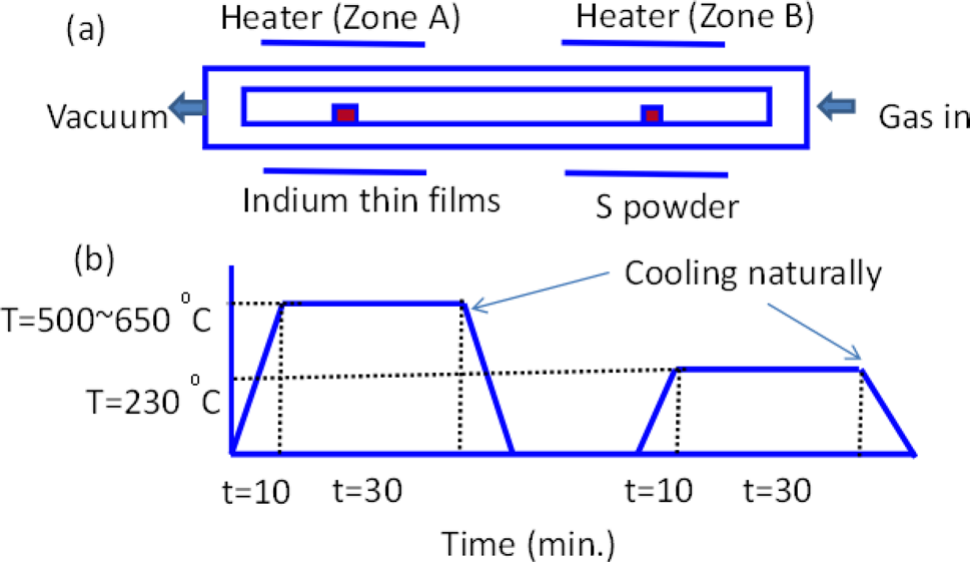
(**a**, **b**): (**a**) The two-zone furnace and (**b**) the temperature–time schedule diagrams for annealing the indium thin films in the sulfur environment at different pressures and temperatures to grow In_2_S_3_ thin films.

The second step: In_2_S_3_ thin films have been grown by chemical vapor deposition method (CVD) using two zone furnaces as presented in Fig. 1a. Indium films are annealed in a sulfur environment under optimized growth conditions including; annealing temperature, pressure, and time. The temperature of the sulfur powder was fixed at 230 °C. While the annealing temperature and pressure of indium films were changed from 500 to 650 °C and from 50 to 200 Torr, respectively. The annealing conditions such as heating/cooling rates, temperature, and time are shown in Fig. 1b. Each sample was annealed for 30 min. During the annealing process, the heating and cooling rates are done in the same environment of sulfur, while the annealing process has occurred at fixed pressure and temperature for every experiment.

The structure and morphology of the thin films have been investigated using X-ray diffraction. The X-ray diffraction patterns were obtained using the diffractometer (Bruker: D8) through (CuKα; λ ~ 1.540 Å). Field emission scanning electron microscopy (FE-SEM), JSM 6400 FE-SEM). The energy dispersive spectroscopy analysis (EDX) was used to check the atomic ratio of In/S for the as-prepared In_2_S_3_ thin films. Photoluminescence (PL) spectra and Raman shifts are measured in a micro-Raman spectrometer in a backscattering configuration excited with an Ar^+^ laser (λ = 514 nm). We have removed the Rayleigh scattering by using a 50/50 beam splitter and two notch filters centered at the laser line. The system is equipped with a single-pass spectrometer with a grating of 1800 grooves/mm and a Peltier-cooled CCD array. The slits are set to an aperture of about 20 μm to provide a resolution of about 0.5/cm. Typical integration times are in the order of 10 s and power in the order of 250 μW to avoid heating effects^20^.

## Results and discussions

### Characterization of synthesized In_2_S_3_ films

To synthesize In_2_S_3_ films, the indium films are deposited by evaporating the indium metal on SiO_2_/Si-substrate. Then, annealing the films in the sulfur environment under optimized growth conditions. The structure and morphology of In_2_S_3_ films will be elucidated by different characterizing techniques including XRD; FE-SEM, PL, and Raman spectroscopy. The overall aim is to be sure that the prepared film is fitted with the proposed structure.

#### X-ray diffraction (XRD)

**Figure 2:**
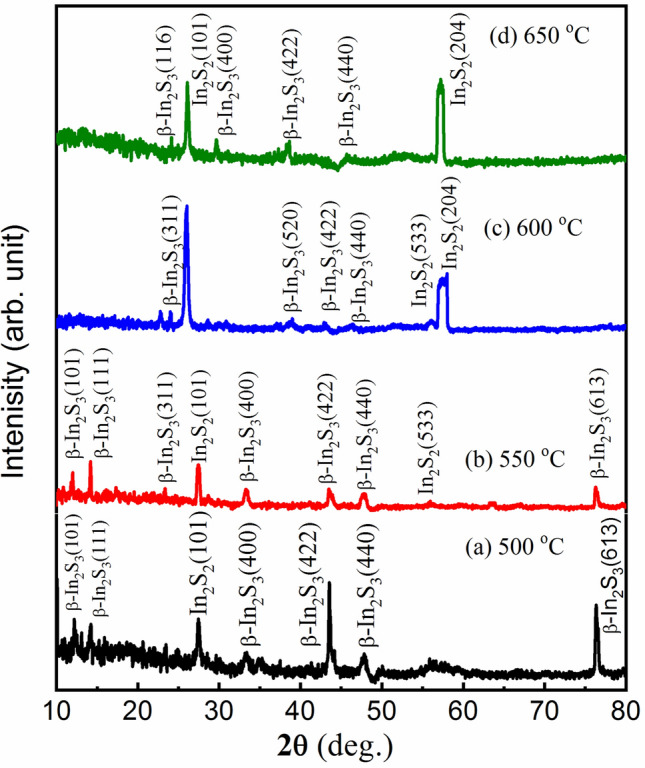
(**a**–**d**). The XRD patterns of the grown thin film of In_2_S_3_ at pressure 100 Torr., and different temperatures (**a**) 500 °C, (**b**) 550 °C, (**c**) 600 °C, and (**d**) 650 °C.

The XRD diffraction patterns of the grown thin film of In_2_S_3_ on the SiO_2_/Si substrate at constant pressure 100 Torr and different temperatures (500, 550, 600, and 650 °C) are presented in Fig. 2a–d. It can be noted that the thin films annealed at 100 Torr is β-In_2_S_3_ and all peaks in panel (a) were indexed to β-In_2_S_3_ structure phase except only one peak at 2θ ≈ 26° is related to (101) In_2_S_2_ phase. Mainly 6 peaks are appearing for two samples annealed at 500 and 550 °C which are related to the β-In_2_S_3_ phase. Panels (c and d) presented the XRD pattern of indium thin films annealed at higher temperatures (500 and 650 °C) with fixed pressure (100 Torr). At the higher annealing temperatures of 600 and 650 °C, there are two new peaks of hkl; (101) and (204) appeared at 2θ = 26° and 57°, respectively. These two peaks are related to the In_2_S_2_ phase structure. Moreover, all peaks related to the β-In_2_S_3_ structure phase decreased and were dominated by the In_2_S_2_ phase structure. This means that the temperature of annealing is high to make sulfide atoms defects. By using this method, the annealing temperature of In_2_S_3_ at a pressure of 100 Torr is nearly 550 °C. This is the 1st step for optimizing the annealing temperature.

**Figure 3:**
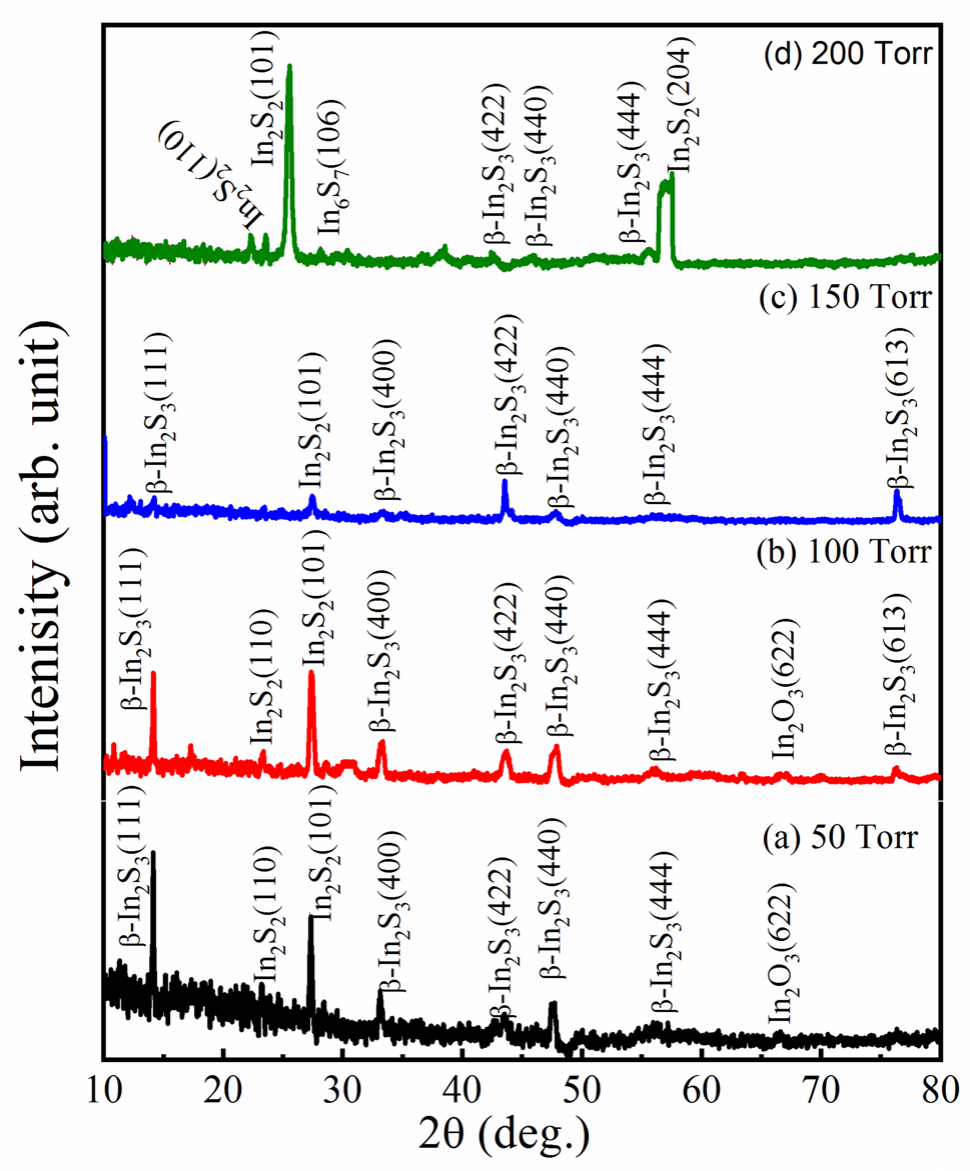
(**a**–**d**): The XRD patterns of the grown thin film of In_2_S_3_ at annealing temperature 550 °C with different pressures (**a**) 50 Torr (**b**) 100 Torr (**c**) 150 Torr, and (**d**) 200 Torr.

Indium thin films have been annealed at a fixed temperature (550 °C) with different pressures (50, 100, 150, and 200 Torr) to study the effect of annealing pressure on the indium thin films. The XRD patterns of the grown thin film of In_2_S_3_ are shown in Fig. 3a–d. As one can be noticed from the experimental data at low pressure (50 Torr), there are 4 peaks. Three of them are related to the β-In_2_S_3_ structure phase and one peak at 2θ ~ 26° is due to the In_2_S_2_ phase structure. With increasing the pressure (100 Torr), the β-In_2_S_3_ structure phase appeared and become very clear. All peaks are indexed to the β-In_2_S_3_ structure phase except for the peak at 2θ ~ 26° is due to the In_2_S_2_ phase structure. This means that the suitable pressure of annealing indium films in the sulfur vapor is around 100 Torr. Moreover, at higher pressures (150 and 200 Torr), the intensities of the peaks related to the β-In_2_S_3_ structure phase decreased and were dominated by the In_2_S_2_ phase structure. The details of XRD pattern diffraction confirmed that the optimum conditions for growing stable β-In_2_S_3_ structure phase thin film using the CVD method are 550 °C and 100 Torr.

#### Field emission scanning electron microscopy (FE-SEM)

**Figure 4:**
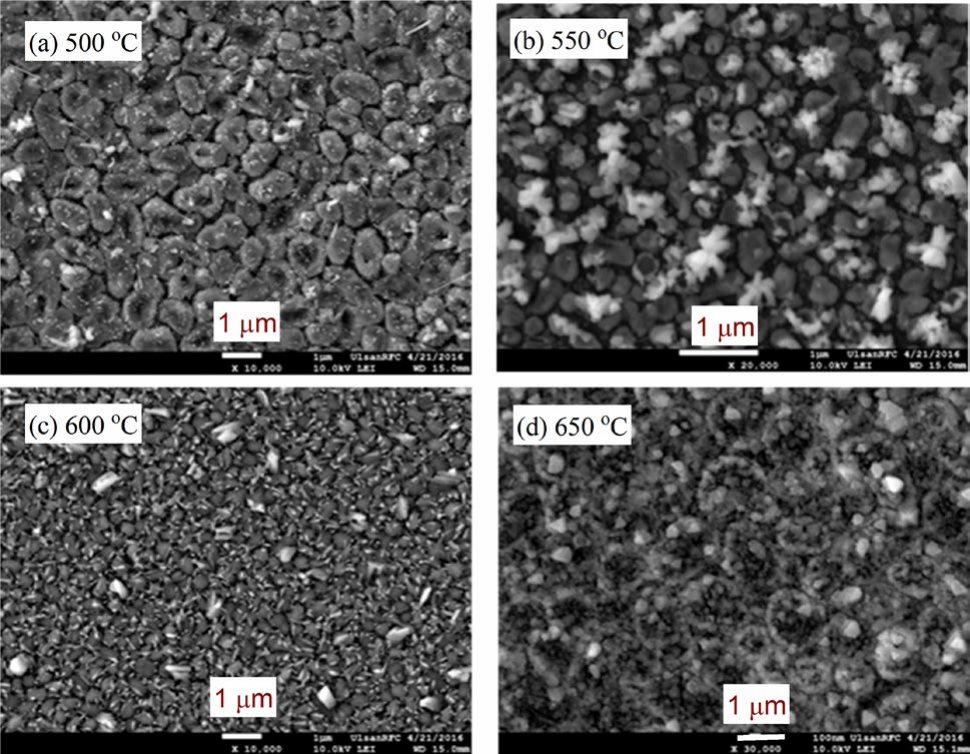
(**a**–**d**): FE-SEM images of the grown thin film of In_2_S_3_ at a pressure of 100 Torr. with different annealing temperatures (**a**) 500 °C, (**b**) 550 °C, (**c**) 600 °C, and (**d**) 650 °C.

Figure 4a–d represents the field emission scanning electron microscopy (FE-SEM) images of the grown thin films of In_2_S_3_ by annealing the indium thin films in sulfur vapor using the CVD method at a fixed pressure (100 Torr) and different annealing temperatures: 500, 550, 600, and 650 °C. The particle size was varied with temperature since the average particle size of the sample annealed at 500 °C is around 98.37 μm. It decreased to 81.16 μm, 45.14 μm, and 47.92 μm with increasing annealing temperatures to 550, 600, and 650 °C, respectively. Besides, the shape of the particles is changed with temperature from a hexagonal shape at 500, 550 °C to a smaller size at 600, and 650 °C. The combination of the experimental data of XRD and FE-SEM confirmed that high pressure (> 100 Torr) and temperature (> 550 °C) changed the β-In_2_S_3_ structure phase.

**Figure 5:**
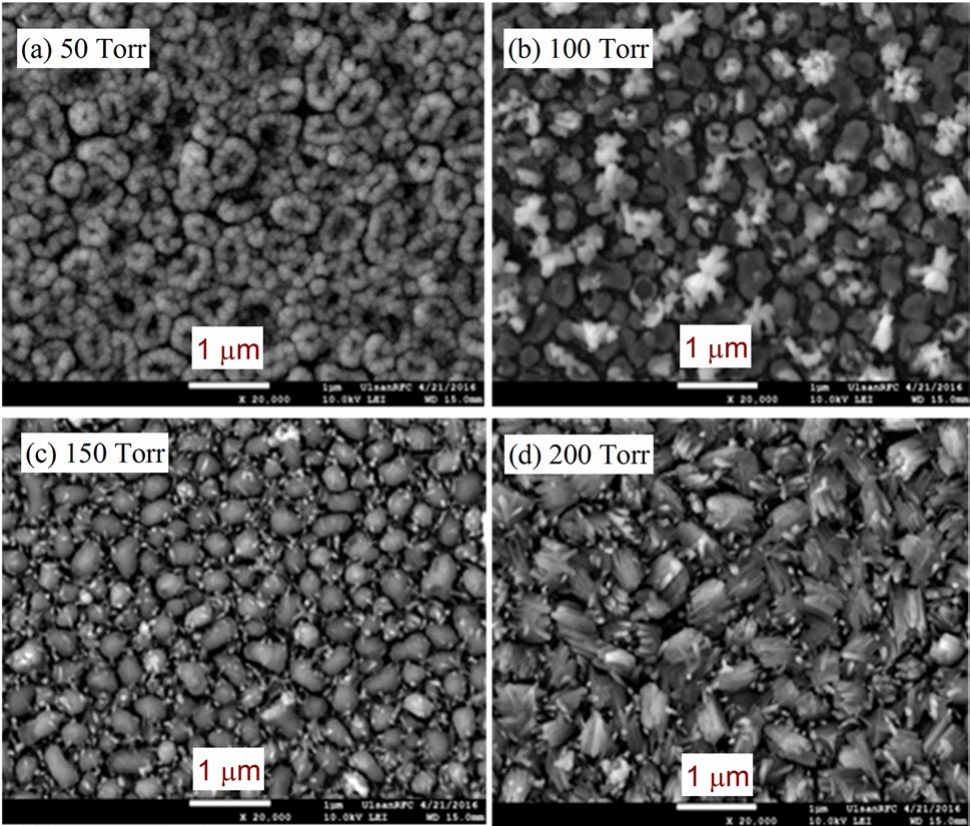
(**a**–**d**): The SEM images of the grown thin film of In_2_S_3_ at temperature 550 °C with different pressures (**a**) 50 Torr (**b**) 100 Torr (**c**) 150 Torr, and (**d**) 200 Torr.

Figure 5a–d depicts the FE- SEM images of the grown thin films of In_2_S_3_ by annealing the indium thin films in sulfur vapor using the CVD method at fixed an annealing temperature 550 °C with different pressures: 50, 100, 150, and 200 Torr. It is clear that at lower pressure (50 Torr) the average particle size (*D*_ag_) was changed and similar to the sample grown at higher temperatures (600 and 650 °C) as observed in Fig. 4. With increasing the pressure from 150 and 200 Torr, the shape and size of the particles are also altred. On the other hand, the particles become clusters at 200 Torr similar to the results identified for higher temperature growing films. Other evidence here is the change in the structure of the as-prepared films by increasing pressure as well as temperatures.

#### Energy dispersive X-ray (EDX) analysis

**Figure 6:**
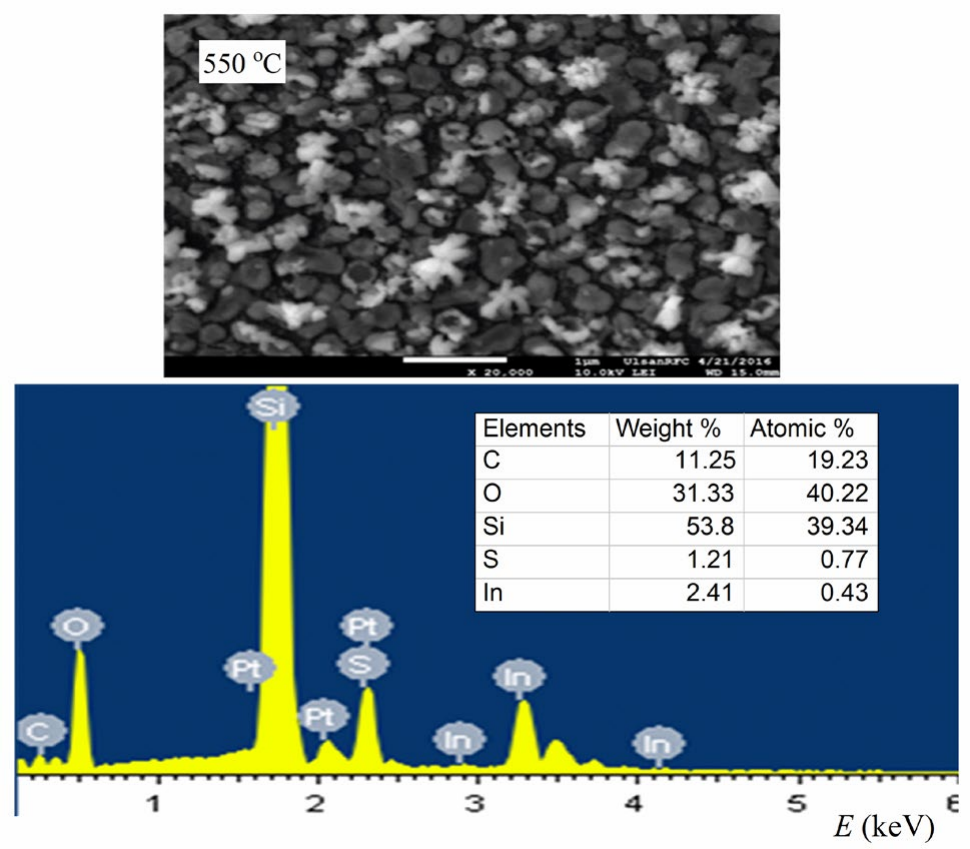
The EDX spectra of In_2_S_3_ thin film at 550 °C and 100 Torr.

Following the FE-SEM analysis, the EDX spectra were studied to confirm the chemical stoichiometry of In_2_S_3_ thin films. The representative EDX pattern was recorded from micro-structures for the sample grown at 550 °C and 100 Torr. As seen from the Table that is displayed in Fig. 6, the atomic ratio of the studied films In: S was found to be 2.12:2.83, i.e. ≈ 2:3. These experimental results revealed that the structures are composed of In, S, C, and O, and the above-mentioned ratio (2:3) is expected for In_2_S_3_. The existence of Si and O in the EDX spectra are related to the (SiO_2_/Si) substrate. Once again, the microstructure study with XRD data, FE-SEM, and EDX confirmed that the optimum conditions for growing stable β-In_2_S_3_ structure phase by CVD method are 550 °C and 100 Torr.

**Figure 7:**
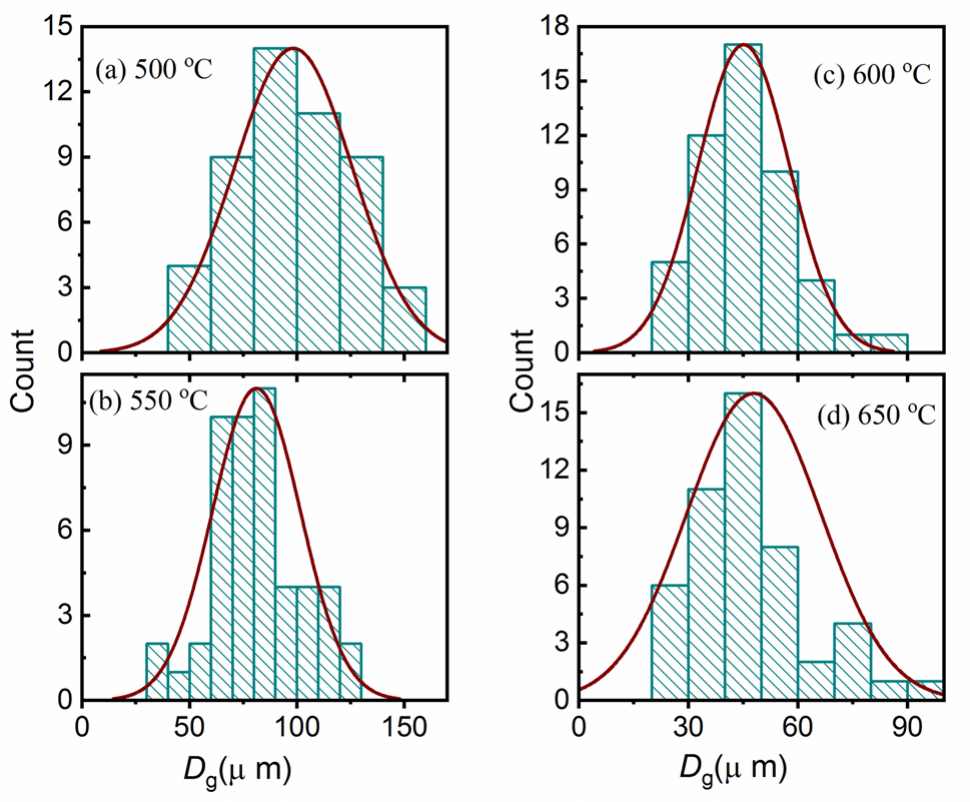
(**a**–**d**): Histograms for the grain size (*D*_g_) distribution of In_2_S_3_ thin films at different annealing temperatures.

The FE-SEM images in Figs. 4 and 5 are used to get the average particle size (*D*_ag_) of the studied thin films by using ImageJ (Fiji) software. This means that the *D*_ag_ was estimated at different annealing temperatures and constant pressure (100 Torr) by using the histograms shown in Fig. 7a–d. Similarly, the values of *D*_ag_ were determined for the studied films at different pressures and a constant temperature (550 °C). All *D*_ag_ values are given in Table 1. As seen from this Table, the values of *D*_ag_ changed with the annealing conditions, i.e., temperature and pressure.

**Table 1:** The average particle size (*D*_ag_) of the as-prepared In_2_S_3_ thin films at different annealing conditions.

Temperature (°C)	*D*_ag_ (μm)	Pressure (Torr)	*D*_ag_ (μm)
500	98.37	50	101.59
550	81.16	100	70.30
600	45.14	150	74.65
650	47.92	200	94.24

#### Raman spectroscopy

**Figure 8:**
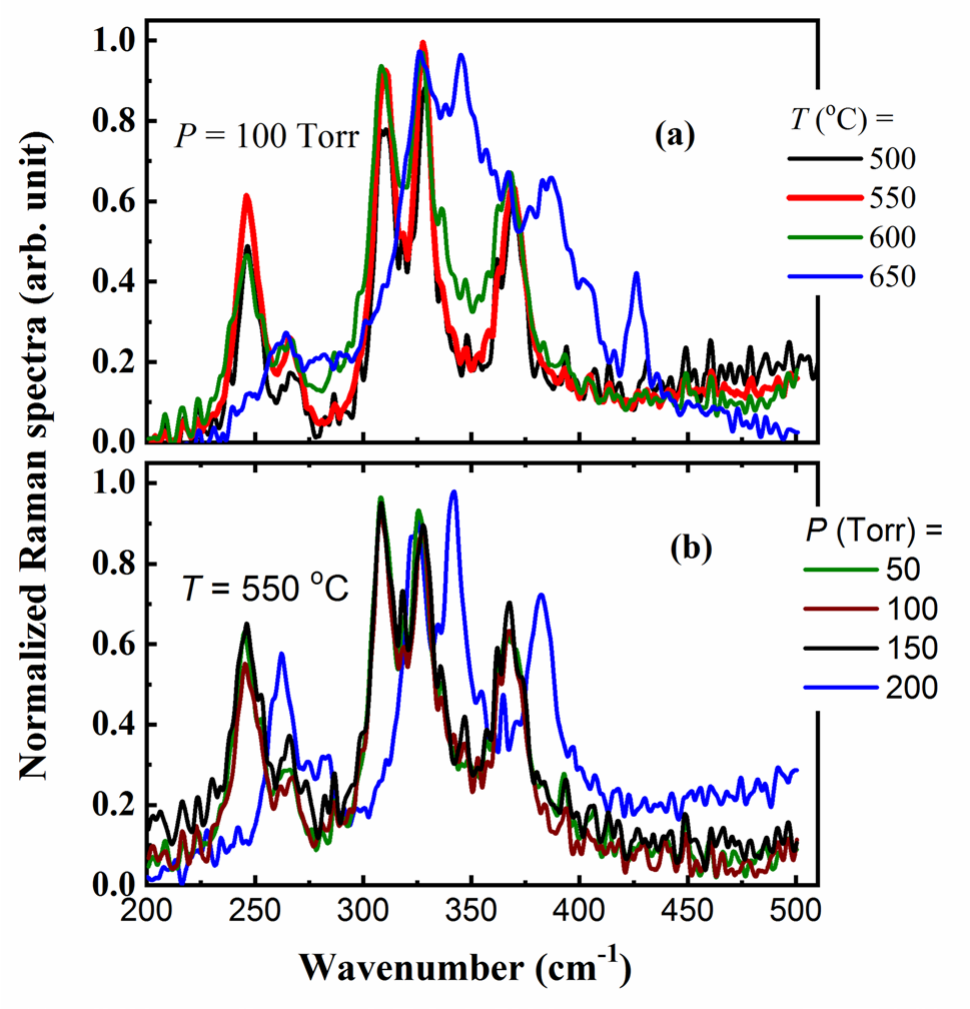
(**a**, **b**): The Raman spectra of In_2_S_3_ (**a**) for samples growing at a pressure of 100 Torr and with different annealing temperatures (500, 550, 600, and 650 °C), and (**b**) the samples with the annealing temperature of 550 °C and with different higher pressures (50, 100, 150, and 200 Torr).

Another possibility to confirm the structure of the studied thin films could be achieved with the help of Raman shift obtained with Raman spectroscopy. Figure 8a,b presents the Raman shifts for the grown β-In_2_S_3_ thin films by annealing the indium thin films in sulfur vapor using the CVD technique with different temperatures and pressures. The spectrum shown in panel (a) the films grown at 100 Torr and different annealed temperatures (500, 550, 600, and 650 °C). Raman spectra for the β-In_2_S_3_ structure phase contain four typical peaks starting at 245, 310, 330, and 365/cm. As seen from the Raman spectra of the grown In_2_S_3_ thin films at higher temperatures 600 and 650 °C (see Fig. 8a), all peaks have been shifted to higher wavenumbers. The shift of the peaks in Raman spectra towards either higher or lower wavenumber could be related to the chemical bond length of molecules. The shorter bond length causes to shift higher wavenumbers and vice versa. Besides, at the annealing temperature 550 °C, the spectra are recorded for samples with different relatively higher pressures as shown in Fig. 8b. It is clear the typical spectra of β-In_2_S_3_ thin films where all peak positions are the same except for 200 Torr. Raman spectra at higher pressure (200 Torr) showed a shift in the peaks of other samples. This result is consistent with that of the XRD and FE-SEM.

**Figure 9:**
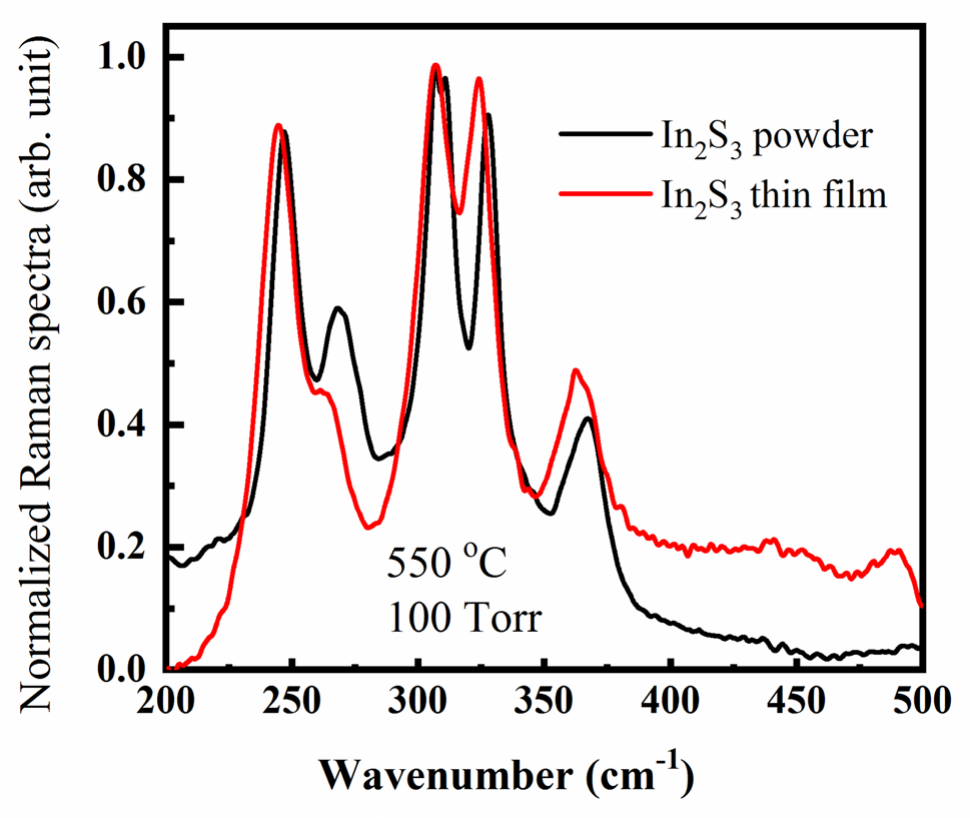
Raman spectra of In_2_S_3_ thin film at a temperature of 550 °C and pressure of 100 Torr. (red color) and powder (black color) as a reference.

It is useful to compare the Raman spectra of powder with thin In_2_S_3_ films. Therefore, Fig. 9 depicts the recorded data of thin film In_2_S_3_ (conditions 100 Torr and 550 °C) and the powder In_2_S_3_. It is observed that the typical spectra are similar to those of the standard spectra of powder and a thin film of the β-In_2_S_3_ structure phase. More evidence is an optimum condition to fabricate the β-In_2_S_3_ thin films is 100 Torr and 550 °C annealing conditions.

#### Photoluminescence spectroscopy

**Figure 10:**
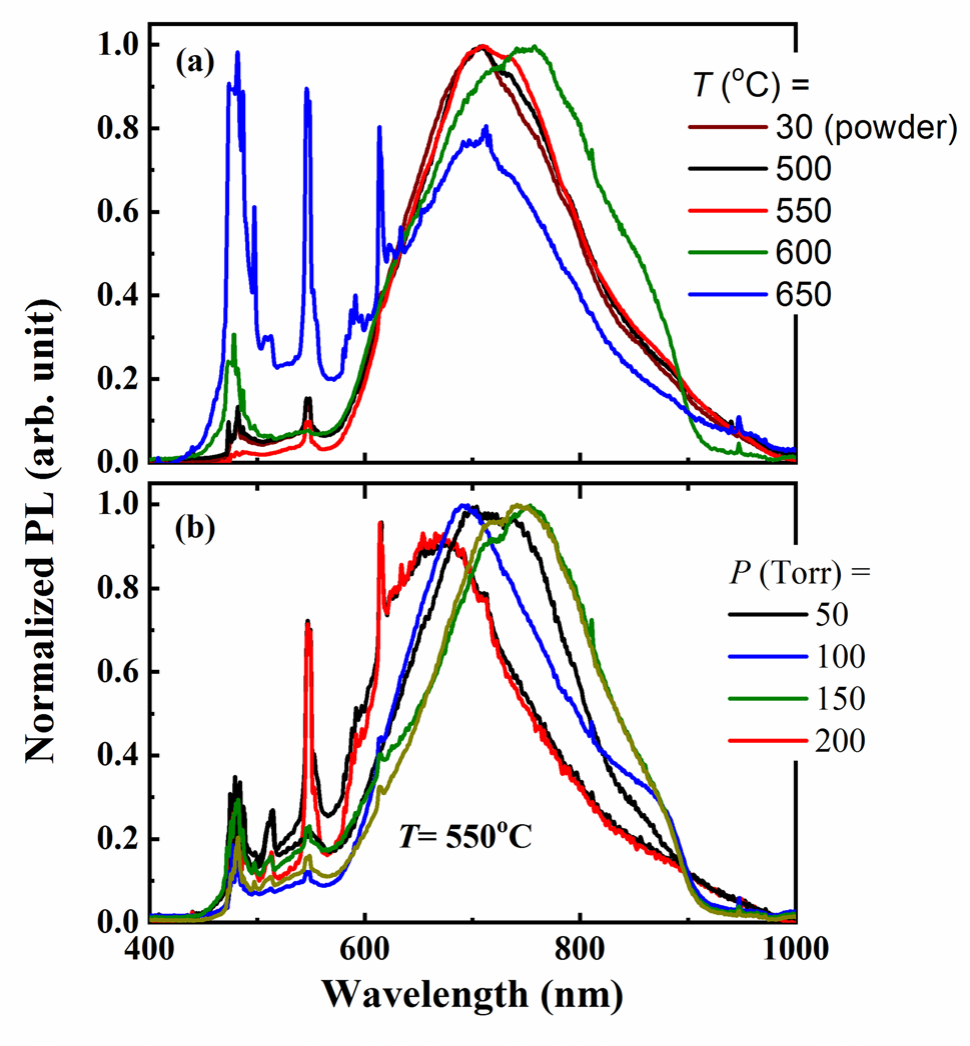
(**a**, **b**). The emission spectra of In_2_S_3_ under 460 nm excitation for samples growing at : (**a**) pressure of 100 Torr with different annealing temperatures (500–650 °C) and (**b**) temperature of 550 °C with different pressures (50–200 Torr).

In Photoluminescence (PL) process, the studied In_2_S_3_ samples absorb the photon of the incident electromagnetic waves and then re-radiate it. This means an excitation for the In_2_S_3_ samples, to a higher energy state followed by a return to a lower energy state photon emissions. Figure 10a displays the PL spectra of In_2_S_3_ samples under excitation at different temperatures. A broad emission band is observed at wavelengths from 600 to 900 nm which can be attributed to the excitonic emission. Similar behavior had been seen in other reported^10,12,18^. As clear in Fig. 10a, the peaks shifted for the thin films annealed in different temperatures starting from 500 to 650 °C. In the experimental data, the powder photoluminescence emission peaks were included for comparison. All curves have similar behavior for all thin films of indium annealed in 500 °C and 550 °C. However, the annealed films at 600 and 650 °C, showed different behavior with peak shifts. Sharp peaks at wavelengths 500, 550, and 600 nm are observed for the annealed 650 °C. Both XRD pattern and FE-SEM images of the sample annealed at 650 °C confirmed the absence of the complete β-In_2_S_3_ phase. On the other hand, Fig. 10b shows the PL spectra of the 550 °C annealed thin film at different pressures. Similar results have been recognized that the increase in pressure shifts the peaks of normalized PL to lower wavelengths. Also, different bands were observed at 200 Torr compared to other studied films.

## Conclusions

The micro-structure data obtained by XRD confirmed the optimum conditions for growing stable β-In_2_S_3_ structure phase by annealing the indium thin films in sulfur vapor using the CVD method at 550 °C and 100 Torr. Moreover, the annealing conditions changed the shape and size of the particles in In_2_S_3_ thin films. The EDX spectra revealed that the chemical ratio of In to S in In_2_S_3_ is about 2:3. Also, the average particle size (*D*_ag_) changed with the change in the annealing conditions. Raman spectra also showed the optimum condition of the thin film In_2_S_3_ fabrication is 100 Torr and 550 °C. When these conditions are changed, i.e., pressure to 200 Torr and temperature to 650 °C, there is a shift in Raman peaks indicating a noncomplete β-In_2_S_3_ structure phase. Besides, photoluminescence (PL) emphasizes the formation of β-In_2_S_3,_ and the optimum conditions are similar to those found by XRD, FE-SEM, and Raman spectra. The outcome results of this work confirmed the optimum condition of the β-In_2_S_3_ structure phase can be obtained using our modified method of preparation to be used in suitable applications.
